# Total hip arthroplasty for intertrochanteric fracture fixation failure

**DOI:** 10.1186/s40001-019-0398-1

**Published:** 2019-12-27

**Authors:** Liyun Liu, Yongqiang Sun, Linlin Wang, Qiankun Gao, Ang Li, Jialin Wang, Yanzheng Gao

**Affiliations:** 1 School of Medicine, Henan University, Kaifeng, 475004 Henan People’s Republic of China; 2 Luoyang Orthopedic Hospital of Henan Province, Orthopedic Hospital of Henan Province, Zhengzhou, 45000 Henan People’s Republic of China; 3 Henan Provincial People’s Hospital, Zhengzhou University People’s Hospital, 7 Weiwu Road, Zhengzhou, 450003 Henan People’s Republic of China

**Keywords:** Elderly patient, Intertrochanteric fracture, Fixation failure, Hip, Arthroplasty

## Abstract

**Background:**

Intertrochanteric fracture is a common fracture suffered by elderly patients. Total hip arthroplasty (THA) is regarded as a salvage operation to restore hip joint function after fixation failure, which remains somewhat controversial due to some clinical potential issues.

**Methods:**

18 elderly patients (average age 70.3 years) each with intertrochanteric fracture fixation failure treated with THA between September 2013 and October 2016 were retrospectively analyzed. Internal fixation treatments involved 5 patients who had received proximal femoral nail anti-rotation, 7 who received locking proximal femur plates and 6 who received dynamic hip screws. All patients were treated with THA using biological acetabular prosthesis and hip arthroplasty (HA) coating skillet femoral prosthesis, with the greater trochanter fixed using wire or steel when necessary. Patients’ Harris scores pre- and post-treatment, SF-36 Health Questionnaire score and digital radiology (DR) were used for joint prostheses initial stability and survival evaluation.

**Results:**

15 patients completed follow-up periods ranging between 19 and 54 months (mean 26.2 months; 1 patient died from a pulmonary embolism, 1 patient died from pulmonary heart disease 1 year after surgery and 1 patient withdrew for personal reasons). There were no joint infections, periprosthetic fractures or dislocations. The average Harris score increased significantly, from 32.68 ± 12.04 points before surgery to 91.08 ± 5.9 points at 24 months post-treatment. SF-36 scores were significantly increased.

**Conclusion:**

THA as salvage treatment for failed internal fixation of intertrochanteric femoral fractures in elderly patients significantly reduced hip pain and restored joint function, and early clinical outcomes were satisfactory.

## Background

Intertrochanteric fracture is one of the most common fractures suffered by elderly patients, and its incidence has been increasing yearly [1, 2]. Among elderly hip fracture patients, intertrochanteric fracture accounts for more than 60–70% of cases with an annual mortality rate of 15–20% [3–5]. The current clinical consensus is to treat intertrochanteric fractures of the elderly with early surgical treatment [6]. Increasing age and osteoporosis present treatment issues due to compromised biomechanical structural strength of the femur. Additionally, internal fixation loosening, cutting, nonunion, coxa vara and other problems may occur following treatment [7–9].

Total hip arthroplasty (THA) is generally regarded as a salvage operation to restore hip joint function as early as possible after fixation failure [10–13]. In this context, however, THA remains somewhat controversial due to potential problems including scar adhesion, blood loss, osteotomy plane angle, partial bone defect, postoperative soft tissue balance, offset reconstruction and postsurgical complications. Here, we describe THA salvage treatment of 18 elderly patients suffering intertrochanteric fracture fixation failure, with positive clinical outcomes.

## Materials and methods

### Patient information and clinical assessment

Patient recruitment was based on the following inclusion criteria: age ≥ 60 years; X-ray diagnosis clearly showing intertrochanteric fracture fixation failure (with features including loosening of internal fixation, fracture or nonunion); no pathologic fracture; patients were able to care for themselves before injury; agree to undergo THA reconstruction surgery; complete clinical records. Exclusion criteria included: age < 60 years; cardiovascular or cerebrovascular disease within the past 3 months; limb abductor less than III grade; unwilling to undergo THA reconstruction surgery.

18 patients in this study were retrospectively analyzed: 5 cases of proximal femoral nail anti-rotation (PFNA) failure (including 2 cases of loosening fracture and 3 cases of femoral cutting); 7 cases of locking proximal femoral plate (LPFP) failure (including 3 cases of loose nail backing, 2 cases of screw channel cutting and 2 cases of plate fracture); 6 cases of dynamic hip screw (DHS) failure (including 3 cases of fracture fixation, 1 case of head and neck cutting and 2 cases of hip varus deformity). 15 patients (8 male, 7 female; age 60–76 years, mean 70.3 years) completed follow-up. All had suffered from hip pain and symptoms including limited activity and shortening of the affected limb (average 3 cm compared to the contralateral limb), and unable to walk with a basic load.

THA reconstruction assessment and surgical implementation were carried out by experienced doctors and nurses. Preoperative evaluation and surgery were performed consistently throughout. All patients received a comprehensive physical examination at admission and detailed records of injuries and medical complications were compiled. Six patients had a history of hypertension, three had diabetes, five had had cerebrovascular disease and one had Parkinson’s disease. We also assessed the condition of affected bone by standard anteroposterior-axial DR and three-dimensional CT reconstruction of the hip. All this work was approved by the Ethics Committee of Orthopedic Hospital of Henan Province.

### Surgical methods

Patients received general anesthesia, were placed in a contralateral supine position and received routine disinfection and a surgical drape. A lateral hip approach was used for LPFP and DHS failure patients, cutting layer by layer along the incision until reaching the greater trochanter. Internal fixation was removed using the original fixed instrument. We also used the lateral hip approach for PFNA failure patients. The 1/3 bundle of the gluteus medius was bluntly dissected longitudinally to its point of attachment, and the attachment points stripped in front of the greater trochanter of the femur. Adhesions were cleared around scar tissue, fully exposing the trochanter and enabling assessment of the greater and lesser trochanters and calcar femorale. If the greater trochanter had an avulsion fracture or was not healed, we directly passed into the joint cavity. If the greater trochanter was intact, we incised the joint capsule to pass into the joint cavity along the front. We fully removed scar tissue around the fractures to evaluate the resected surface of the femoral calcar. Following osteotomy the acetabulum was exposed and progressively filed until the acetabulum and acetabular wall bled. The acetabular cup was maintained at an abduction angle of 35°–45°; the polyethylene liner was maintained at an anteversion angle of 15°–20° while the ceramic liner was maintained at an anteversion angle of 25°. Two cup screws were applied to increase initial stability. Maintaining limb adduction with external rotation and hip flexion to fully reveal the proximal femoral canal, the canal was milled in a 15° forward direction (in the same plane as the femoral condyles) and an appropriate prosthesis was embedded. Maintaining the center of the femoral head of the prosthesis level with the tip of the greater trochanter and then resetting the hip we evaluated whether the reset was difficult or the joint was stressed. If so, we performed a second release of surrounding soft tissues and removed scarring. A nonhealing greater trochanter was rebuilt and fixed, using a tension band to fix smaller fragments and a memory alloy hook plate for larger fragments. The incision was flushed and a suction drainage tube placed after closure. 5 patients were treated using proximal fixation prosthesis (APLHA porous-coated biological handle, Smith & Nephew) and 9 patients were treated by distal fixation of the prosthesis (Solution biological stem, DePuy) due to osteoporosis or severe deformity of the proximal femur.

### Postoperative treatment and follow-up

Surgical duration and blood loss were recorded. Antibiotics were administered 24 h after surgery and body water and electrolyte balance were maintained. Quadriceps contraction exercises were begun 2 days after surgery. Ambulation with a mobility aid was allowed 7 days after surgery. On day 3 post-surgery patients underwent standard hip anteroposterior and axial DR. Patients received follow-up DR at 3, 6, and 12 months. Harris hip score and SF-36 Health Questionnaire scores were recorded to evaluate initial stability of the joint prosthesis and limb recovery. A Harris score of ≥ 90 points corresponds to excellent; 80–89, good; 70–79, tolerable; < 70, inferior [14]. SF-36 scores account for physical function (PF), role physical (RP), general health self-assessment (GH), social function (SF), bodily pain (BP), vitality (VT), mental health (MH) and role emotional (RE) [15].

### Statistical analysis

Data were analyzed using SPSS20.0 statistical software and expressed as ${\bar{\text{x}}} \pm {\text{s}}$. Patient follow-up scores were assessed using the paired *t* test. *P* < 0.05 was considered statistically significant.

## Results

Surgery was successful in all cases. In one case the femoral trochanter split intraoperatively and was tied with steel wire and fixed with a tension band. In another case intraoperative difficulty arose from subtrochanteric osteotomy, which was addressed using 1/3 tubular plate fixation. Intraoperative blood loss was 250–1200 ml (average 550 ml). The duration of surgery was 90–170 min (average 100 min).

**Fig. 1:**
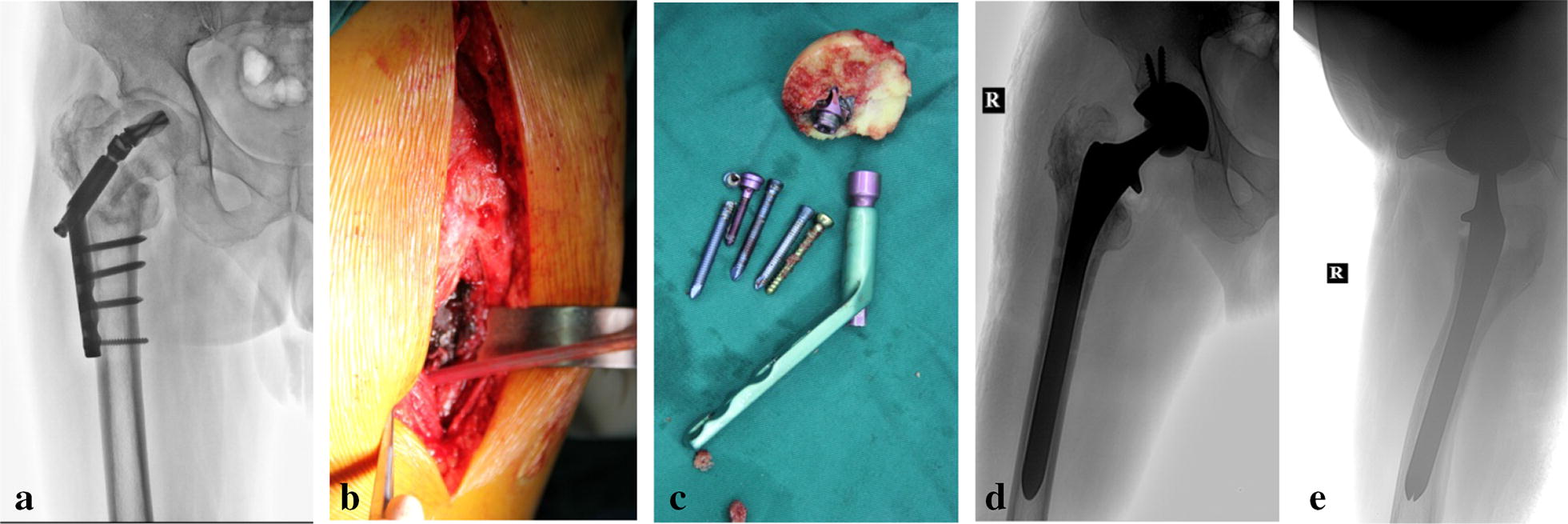
**a** A 64-year-old male patient underwent open reduction and internal fixation (DHS) surgery for a trauma-related intertrochanteric fracture of the right femur. At 19 months post-surgery the patient exhibited nonunion and varus deformity of the hip. **b** Surrounding soft tissue and hyperplastic callus released during surgery. **c** DHS internal fixation removed. **d** Hip digital radiology (DR) at 3 months showing successful THA reconstruction. Hip joint center and eccentricity were well reconstructed. **e** Postoperative lateral hip DR

**Fig. 2:**
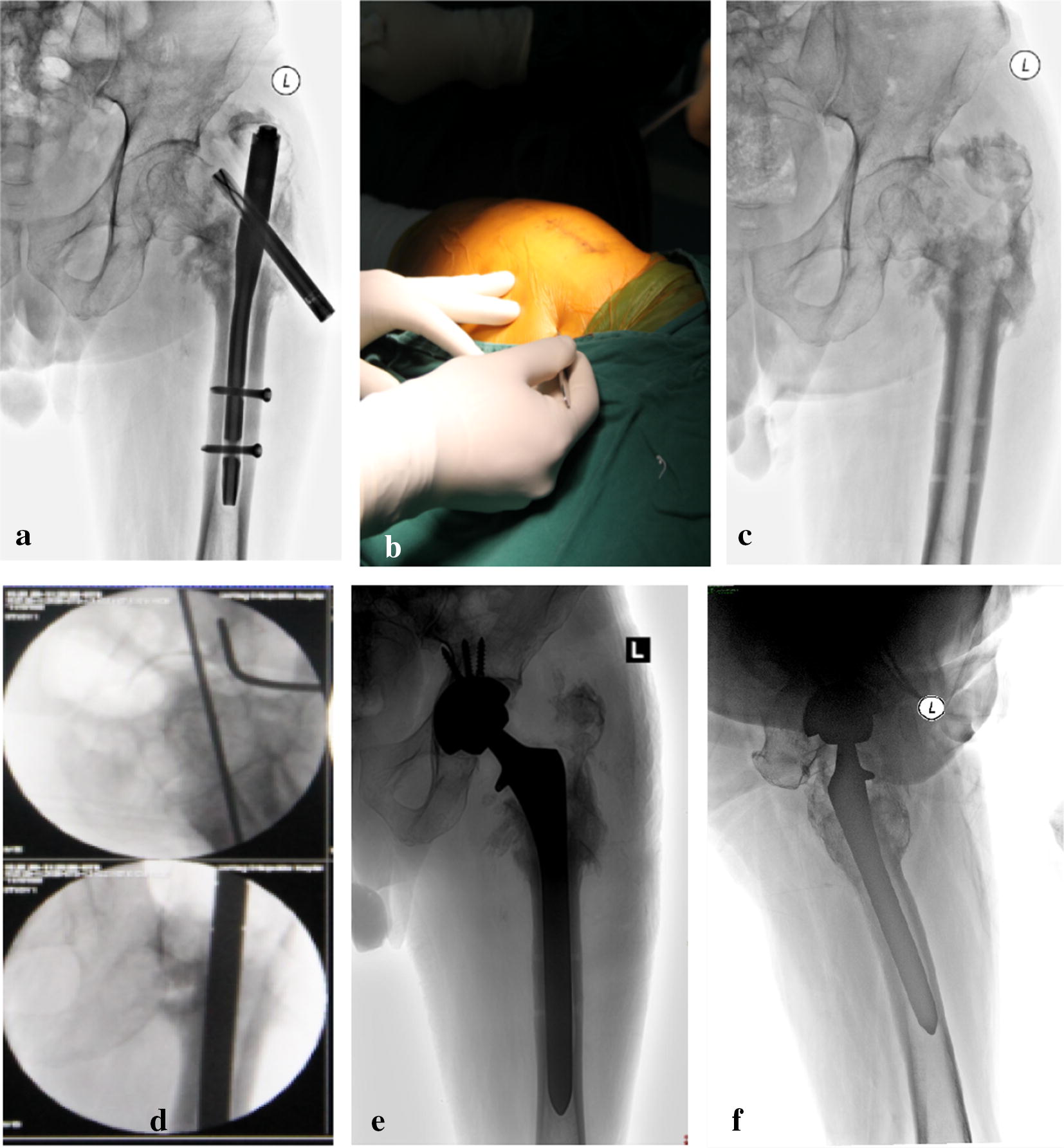
**a** A 71-year-old male patient underwent closed reduction and internal fixation with PFNA for a fall-related intertrochanteric fracture of his left femur. At 16 months post-surgery the patient exhibited emerging fracture nonunion and loosening. **b** Intraoperative incision site. **c** Intraoperative anteroposterior DR of hip joint after PFNA. **d** Intraoperative Kirschner canal filing. **e** Postoperative hip DR. **f** Postoperative lateral hip DR

One patient died 1 month after surgery as a result of an acute pulmonary embolism. Another patient was found to have a deep vein thrombosis 3 months after surgery, which was improved after treatment. 16 patients without serious medical issues were discharged after their wounds had healed. One patient died 6 months after surgery due to pulmonary heart disease and was lost to follow-up. Another patient subsequently dropped out of the study for personal reasons. A total of 15 patients (8 male and 7 female), age 60–76 years (mean 70.3 years) were followed up through completion of the study. The duration of follow-up was 19–54 months (average 26.2 months). Pain symptoms disappeared for the majority of patients. Three patients noted hip discomfort following prolonged activity that disappeared after resting. There were no occurrences of joint infection, periprosthetic fracture, prosthesis dislocation or other postsurgical complications (Table 1). The average Harris hip score increased from 32.68 ± 12.04 (range 10–59) preoperatively to 91.08 ± 5.9 points (range 56–99) at 24 months follow-up (Table 2). SF-36 Health Questionnaire scores suggested that total hip replacement surgery could significantly lower risk of hip pain, promote hip functional recovery and improve overall health and quality of life (Table 3). Figures 1 and 2 illustrate typical cases.

**Table 1:** Postoperative complications of total hip arthroplasty in 18 patients

Complications	1 month postoperation	3 months postoperation	6 months postoperation	12 months postoperation
Pulmonary embolism	1	0	0	0
Infections	0	0	0	0
Periprosthetic fracture	0	0	0	0
Vein thrombosis	1	0	0	0
Prosthesis dislocation	0	0	0	0
Pulmonary heart disease	0	0	1	0

**Table 2:** Harris scores during follow-up

Time (after operation)	Number of cases	Harris points (${\bar{\text{x}}} \pm {\text{s}}$)			Good rate (%)
		Preoperation	Postoperation	*P* values	
3 months	15	32.68 ± 12.04	80.52 ± 3.1	0.00	73.3
6 months	15	32.68 ± 12.04	86.38 ± 4.7	0.00	80.0
12 months	15	32.68 ± 12.04	90.16 ± 5.2	0.00	86.7
24 months	15	32.68 ± 12.04	91.08 ± 5.9	0.00	86.7

**Table 3:** SF-36 Health Questionnaire patient scores

Aspect	Preoperation	24 months postoperation	*t* value	*P* value
Physiological function (PF)	29.6 ± 3.4	72.3 ± 12.1	16.2	0.00
Role physical (RP)	22.1 ± 8.4	73.0 ± 19.8	12.4	0.02
General health (GH)	17.5 ± 6.2	68.6 ± 13.1	19.7	0.00
Social function (SF)	26.7 ± 14.0	73.3 ± 15.8	14.9	0.04
Bodily pain (BP)	29.0 ± 13.6	76.8 ± 12.7	15.1	0.00
Vitality (VT)	20.5 ± 5.3	74.0 ± 10.7	28.3	0.01
Mental health (MH)	17.2 ± 4.8	76.1 ± 8.2	32.9	0.00
Role emotional (RE)	24.1 ± 15.6	76.8 ± 21.0	10.6	0.01

## Discussion

Intertrochanteric fracture is a common fracture injury among the elderly, the incidence of which has been steadily increasing [16]. Conservative treatment approaches can result in long periods in bed, which in elderly patients commonly suffering from multiple disorders can increase the occurrence of medical complications and also easily lead to lower limb shortening and varus. Surgical treatment for intertrochanteric fracture in the elderly has recently become a trend [17]. Some experts contend that elderly intertrochanteric fracture should be treated by total hip arthroplasty in the first instance, allowing early ambulation exercise to reduce the incidence of prolonged bed rest-associated complications. Elderly intertrochanteric fractures, however, can be associated with numerous problems including severe crush fracture, local osteoporosis and bone defects. Such fractures are also prone to primary poor stability of the prosthesis, loosening subsidence and other complications following total hip arthroplasty [18, 19]. For these reasons internal fixation is considered the most effective treatment for senile intertrochanteric fractures and is recommended as such by the majority of clinical orthopedic surgeons [20].

Currently, the most common approaches to internal fixation are extramedullary fixation (representative of DHS and LPFP) and intramedullary fixation (representative of Gamma nail and PFNA). Clinical efficacy has been improved by strict regulation of technical operations and surgical indications [21, 22]. Cronel et al. believe that the use of DHS surgery has long been widely accepted by orthopedic surgeons if there are no serious surgical contraindications. DHS is an extramedullary internal fixation system, characterized by firm fixation, sliding pressurization, and low cost. However, this surgery is more traumatic. DHS is fixed on the lateral cortical bone of the femur with longer arm is longer and larger bending distance. If the medial cortical bone has defects, stress concentration is likely to occur, resulting in some complications such as medial cortex compression, nail cutting, loose screws and even fractures of steel plate, in addition to poor anti-rotation ability and intramedullary varus. Especially, in the reverse obliquity intertrochanteric fractures, the fixation failure rate can reach 24–56%. Among patients recruited to the current study were 13 cases with extramedullary fixation LPFP/DHS failure including 3 with internal fixation loosening, 3 with cutting of femoral head, 5 with fixation fracture and 2 with varus deformity. There were 5 cases of intramedullary fixation of PFNA failure, including 3 cases with loosening or fracture and 2 cases with femoral head cutting. Our analysis indicated a number of reasons for fixation failure. First, incorrect indications for internal fixation in the case of comminuted unstable fractures. In such cases stress cannot be conducted across the calcar femorale, which is prone to stump rotation and screw loosening that cuts the femoral head leading to fixation fatigue fracture. According to Zhang et al., a dynamic hip screw can cause secondary mechanical damage of unstable intertrochanteric fractures [23]. Second, because of poor fracture fixation, inside bone support is lacking and the distal femur does not conduct stress. The excessive, concentrated stress leads to varus deformity. Third, severe osteoporosis led to the screw securing less bone mass in the head and neck. Combined with early activity this can result in a nail back. Kim et al. report that osteoporosis is one of the key factors affecting DHS fixation [24]. Therefore, we consider extramedullary fixation only for stable intertrochanteric fractures. However, for elderly patients with peritrochanteric fractures, especially those with osteoporosis, internal fixation has high rate of biomechanical failure. Due to insufficient bone mass, low retention force of internal fixation on bones, it is hard to achieve stable bone contact within the fracture block even if the medial and posterior cortical contacts are restored intraoperatively. Therefore, DHS internal fixation should be used with caution for unstable intertrochanteric fractures, especially in the elderly patients with osteoporosis. In contrast, minimally invasive procedures by using intramedullary fixation are more realistic for elderly patients with osteoporosis, which may help these patients decrease the incidence of hip varus, improve self-confidence, return activities earlier, and as a result reduce the burden of family escort. Fourth, poor fixation location with more screw concentrated in the top of the head and neck where bone is thin and therefore prone to cutting. Finally, early postoperative weight-bearing and progressive limb functional training were not adjusted for individual patient differences and led to internal fixation failure.

Fixation failure after surgery to treat elderly intertrochanteric fracture is a serious complication. The long-term bed rest can worsen osteoporosis and render complications deadly. At the present time some controversy exists regarding treatment of intertrochanteric fracture postsurgical fixation failure. Controversial factors concern patient age, type of fracture, osteoporosis, etc. Younger patients frequently undergo full re-fixation and bone grafting or change to intramedullary fixation due to better local bone and cartilage because the head and acetabulum are normal. Elderly patients often have severe local osteoporosis and greater bone loss that results in poor clinical efficacy from fixation renovation and bone grafting. Here, a semi-artificial hip or total hip arthroplasty is feasible and permits early ambulation and minimized risk of complications from bed rest. Haidukewych et al. evaluated 44 elderly cases of failed internal fixation of intertrochanteric fractures undergoing artificial joint replacement therapy. Hip pain disappeared or was significantly reduced after surgery, and function improved significantly [25]. A 5-year follow-up of 42 patients with intertrochanteric fracture fixation failure who underwent THA showed a success rate of 99% [26]. Among 21 elderly hip fracture fixation failure cases receiving artificial joint replacement, Harris scores increased from 32 points (preoperative) to 79 points at 1-year follow-up [27] and scores showed additional significant improvement at 2.3 years.

Here, we studied 15 cases with complete follow-up. Harris hip scores were significantly improved at 24 months. Pain symptoms disappeared for most of the patients. No patients suffered joint infection, periprosthetic fracture, prosthesis dislocation, deep vein thrombosis or other complications following surgery. This indicated that artificial total hip arthroplasty restored the hip joint function for the intertrochanteric fracture fixation failure in elderly patients, and increased the score of hip activities. Of note, our study introduced the SF-36 Health Status Scale into the postoperative evaluation system. The SF-36 Health Status Scale has advantages of quick response and a wide range of applications suitable for many types of patients. It can be used to evaluate quality of life for patients after THA and is noted for reliability, validity and responsiveness. We combined Harris score and SF-36 Health Questionnaire to evaluate postoperative recovery of hip function and quality of life.

Preoperative assessment, an appropriate surgical plan and perioperative management are especially important in treating elderly patients prone to internal disease, surgical trauma and high surgical risk. For these reasons, clinical practice should include improved preoperative assessment with full consideration of disease complications, anticipation of likely surgical complications, and efforts to minimize the duration of surgery, the amount of blood loss and the risk of surgical trauma. Postoperatively, a personalized rehabilitation program should be developed for each patient, and patients encouraged to initiate early ambulation with the appropriate protective safeguards in place. Such measures may significantly improve the general condition of the patient. It should be noted that our study has some limitations. First, the number of cases was relatively small due to low incidence of intertrochanteric fracture fixation failure in the elderly patients. Second, there was no control group in the present study. A large-scale case–control study would be beneficial for statistical analysis when using THA, and help expand the use of THA in the treatment of femoral intertrochanteric fixation failure in the elderly patients.

## Conclusion

THA as a salvage treatment for fixation failure is gradually being embraced by the majority of clinical orthopedic surgeons for elderly intertrochanteric fracture patients. It can significantly reduce the risk of early hip pain and improve function and patient quality of life. Further clinical follow-up studies will be required to confirm positive long-term outcomes.

## Data Availability

The datasets are available under reasonable request.
